# Evaluation of Hair Characteristics and Animal Age on the Impact of Hair Cortisol Concentration in Feedlot Steers

**DOI:** 10.3389/fvets.2019.00323

**Published:** 2019-09-24

**Authors:** Faith Baier, Temple Grandin, Terry Engle, Lily Edwards-Callaway

**Affiliations:** Department of Animal Sciences, Colorado State University, Fort Collins, CO, United States

**Keywords:** animal welfare, cattle, cortisol, hair, stress

## Abstract

Hair cortisol is a novel biomarker of chronic stress. The objective of this study was to determine the effect of hair color and length, as well as, animal age on hair cortisol concentration in beef feedlot steers. Nineteen beef crossbred steers used for nutrition research and housed in a small feedlot setting were used for this study. Seven of the steers (680 ± 4.5 kg; ~9 years of age) were fitted with ruminal fistulas and duodenal cannulas. The other 12 steers (473 ± 3.1 kg; ~2.5 years of age) were fitted with only ruminal fistulas. Hair samples from each steer were collected throughout a period of 6 weeks from six different areas and analyzed for cortisol concentrations. One pre-determined area was shaved each week for 5 weeks (Weeks 1–5). During week 6, all five, previously shaved areas and an additional area was shaved to collect hair samples of various lengths. Hair length was recorded prior to the collection of each hair sample. Only data from the last week (Week 6) of collection were included in the analyses. Steers were categorized into one of three groups: old with black hair (OB, *n* = 3); old with white hair (OW, *n* = 3); young with black hair (YB, *n* = 12). Older steers exhibited greater hair cortisol concentrations than younger steers (*P* < 0.001). Hair cortisol concentration was not impacted by duration of growth (*P* = 0.33). Cortisol concentrations exhibited a weak, positive correlation with hair length (*r* = 0.33, *P*-value = 0.01). The average hair growth per week of beef steers in the winter months was calculated to be 0.90 mm. Further research should be performed to improve our understanding of the effect of hair characteristics, sampling methodologies and analysis techniques on hair cortisol concentrations.

## Introduction

Physiological parameters are often used to quantify animal well-being. Cortisol is commonly measured to assess stress, whether acute or chronic, in many livestock species. This hormone can be measured using several different mediums—blood (1, 2), saliva (3, 4), urine (5), and feces (6). These parameters provide an acute, time-point measurement of cortisol concentration (7, 8). The measured cortisol concentration when using these methods, specifically blood, can be affected by several factors, such as circadian rhythms (9), handling (10), restraint (11), and degree of habituation or acclimation (12).

Hair cortisol has potential to be a useful non-invasive, measurement tool of chronic stress (13). Measuring cortisol in hair is thought of as a stressful event “retrospective calendar” representing a chronic or long-term period (14). The use of this medium has been explored in a multitude of species, including but not limited to rock hyrax (15), rhesus macaques (16), domestic dogs and cats (17), sows (18), sheep (19), humans (20), and coyotes (21). Studies have been performed with beef (22) and dairy cattle (23, 24); however, results have shown varying conclusions related to cattle. Factors, such as hair color (25, 26), collection method (22), sampling location (22, 25), age (24), pregnancy (27), season and weather change (28) have been shown to affect cortisol concentration in hair to various degrees in cattle. Specifically related to beef cattle, hair cortisol measurements have been used to quantify stress related to castration (29, 30), excitability (31), and long-distance transport (32, 33).

A relatively, small amount of research has been performed with hair cortisol quantification in beef cattle. The objective of this study was to determine the effect of hair characteristics (hair color and length) and animal age on hair cortisol concentration in beef feedlot steers.

## Materials and Methods

### IACUC Protocol

Prior to the initiation of this study, animal use and associated procedures were approved by the Colorado State University (CSU) Institutional Animal Care and Use Committee (Protocol #'s 16-6550A and 17-7107A).

### Animals

This preliminary study was conducted on 19 beef crossbred steers over the period of 6 weeks in the months of December 2017 and January 2018. The animals were housed in a small feedlot setting at CSU's Agricultural Research, Development, and Education Center (ARDEC, Fort Collins, CO) and fed a diet that met all NRC (34) requirements. Seven of the steers (680 ± 4.5 kg; ~9 years of age) were fitted with ruminal fistulas and duodenal cannulas (at 14 months of age). The other twelve steers (473 ± 3.1 kg; ~2.5 years of age) were fitted with ruminal fistulas only (at 18 months of age). The fistula surgery for these twelve steers took place in September 2017. The surgeries for the 9-years-old steers took place in 2012. All fistulas were implemented for the purposes of nutrition research, not this particular study. Old steers with both a rumen fistula and duodenal cannula were housed together in two combined 10 head pens (12 × 61 m). Young steers with only a rumen fistula were housed together in a pen of the same dimensions. One of the old steers with both a ruminal fistula and duodenal cannula was humanely euthanized during the second week of the study due to health concerns.

### Animal Handling

All animals were subjected to the same handling techniques and sampling methods. All samples were collected within the same timeframe each week during a once weekly 7–9 a.m. time period. Data were collected during normal routine handling for fistula cleaning to avoid creating additional stress. Animals were restrained in a hydraulic chute (Silencer, Silencer Hydraulic Chutes, Stapleton, NE) using low stress handling through a serpentine crowd pen and chute design.

### Experimental Design

**Figure 1:**
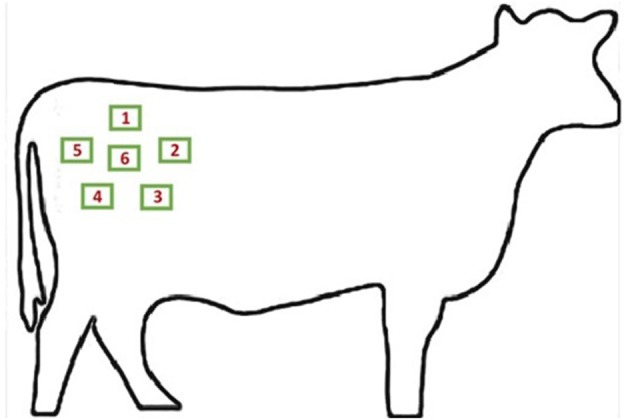
Diagram of hair sampling areas in the right rump region. Each individually labeled green box corresponds with a sampling site measuring ~2.54 × 6.35 cm in area. Image source: http://clipart-library.com/outline-of-a-cow.html.

**Figure 2:**
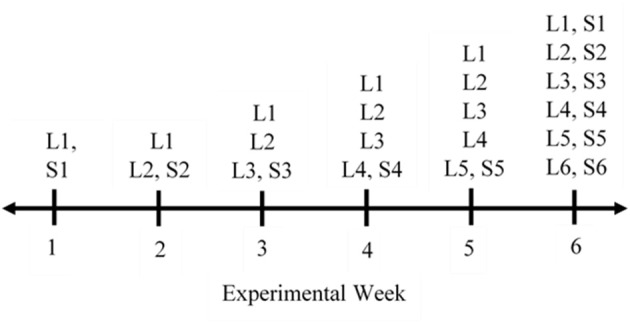
This timeline graphic outlines the measurements taken over the period of 6 weeks for each steer. Data collected are defined as follows: L# (1–6), hair length for the specified area was measured in the rump region; S# (1–6), hair sample for the specified area was collected.

Hair samples and hair length measurements were collected over a period of 6 consecutive weeks. All steers were categorized into one of three cattle groups: old with black hair (OB, *n* = 3); old with white hair (OW, *n* = 3); young with black hair (YB, *n* = 12). Old steers were ~9 years of age and young steers were ~2.5 years of age. No steers fit the demographic of young with white hair. All steers had solid colored hides; therefore, each hair sample consisted of only one color—white or black. Data collection occurred during the same day of each week. Each week, pre-determined areas on the right rump of each animal were shaved (Figure 1). Multiple measurements and samples were collected from each animal depending on the sampling week (Figure 2). During week 1, the designated area number one was shaved on each animal; during week 2, the designated area number two was shaved on each animal continuing with the same sampling scheme throughout week 5. On the last collection day during week 6, all six areas were shaved—five areas (S1–S5) were re-shaved and one area (S6) was newly shaved. Specific areas were shaved at variable times to collect hair samples at different lengths (L1–L6). In total, 11 samples were collected from each steer. Only the six samples from each steer collected during week 6 were included in the data analysis as these were the samples that displayed different lengths of hair for each steer over specific growing periods.

### Hair Measurement and Collection

Hair length measurements were taken at multiple time points to collect hair measurements at different lengths over the course of the 6-weeks experimental period. Due to the poor functionality of digital calipers in cold temperatures, a transparent ruler (Golden Harvest Seeds, Minnetonka, MN) was used to measure hair length for weeks one through five. For week 6, a calibrated, digital caliper (Neiko 01407A, Neiko Tools, Taiwan) was used to measure the hair length of each animal, along with the same previously used ruler for consistency. Measurements of hair length were recorded for the appropriate area on the rump region, pertaining to location and experimental week. All caliper measurements were obtained and verbally read to another researcher for data recording. The hair color of the animal was also recorded.

After length measurement, hair samples were collected from each animal using a cordless livestock clipper (Andis Pro Clip Pulse Ion, Andis Company, Sturtevant, WI) fitted with an adjustable, detachable No. 40 blade. Small, square areas of hair, ~2.54 × 6.35 cm, were shaved from the rump region on the right side of the steer. The hair was shaved as close to the skin as possible. The clipper was cleaned and disinfected with 70% isopropyl alcohol (Equate, Bentonville, AR) between animals. Each hair sample was immediately placed in an individually labeled Ziploc® bag and stored in an opaque container. The cover of the container was kept closed during sample collection except to place the most recently collected hair sample into the container. Hair samples were stored in an opaque container in Ziploc® bags at room temperature until analysis to reduce exposure to light (29). A total of 198 samples were collected over the course of the 6-week period.

### Lab Analysis

Hair samples from week 6 of collection were used for analysis, representing a total of 108 samples with six samples from each steer. Each hair sample was removed from the plastic bag and cut into small fragments (~5 mm) using scissors. The scissors were cleaned with methanol (Optima LC-MS grade, Fisher Scientific, Waltham, MA) after each sample. Hair was submerged in liquid chromatography mass spectrometry (LC-MS) grade water (Optima LC-MS grade, Fisher Scientific, Waltham, MA) and placed on a shaker (Reliable Scientific Rocking Shaker, Reliable Scientific Inc., Hernando, MS) for 4 h at 37°C. To dry the hair, acetone (Optima LC-MS grade, Fisher Scientific, Waltham, MA) was added, and samples were shaken at room temperature for 2 min. Next, samples were placed under a stream of nitrogen gas until completely dry. Cleaned, dry hair was weighed (20 ± 0.5 mg) into a 2.0 mL glass vial. Then, 1.5 mL of the internal standard methanol (100% methanol spiked with d4-Cortisol at 26.67 pg/mL) was added. Samples were sonicated for 16 h at 50°C in a covered bath sonicator (Branson Ultrasonic Cleaner, Branson Ultrasonics, Danbury, CT). After sonication, hair was pelleted to the bottom of the tube via centrifugation (Model 5430, Eppendorf, Hauppauge, NY) at 3,000 × g for 20 min. The methanol supernatant was transferred to a 1.5 mL to microfuge tube and incubated at −80°C for 1 h to precipitate out proteins and other insoluble particulates. The samples were centrifuged at 18,000 × g for 20 min at 4°C. The supernatant was transferred again to a new tube and dried under nitrogen gas. Samples were resuspended in 100 uL of 100% methanol. Resuspended material was vortexed and centrifuged again at 18,000 × g for 5 min 4°C. Finally, 80 uL of resuspension was transferred to a LC-MS vial insert. To make a representative quality control (QC) sample, 10 uL volume of each final extract was pooled together and aliquoted into three separate QC vials.

Sample analysis via liquid chromatography tandem mass spectrometry (LC-MS/MS) was performed using a Waters Classic Acquity UPLC coupled to a Waters Xevo TQ-S triple quadrupole mass spectrometer (Waters Corporation, Milford, MA). The calibration curve was constructed using washed test samples of cattle hair prepared as described above. A surrogate “light” standard of 13C-Cortisol (Sigma-Aldrich, Inc., St. Louis, MO) was used, while the d4-Cortisol (Cerilliant Corporation, Round Rock, Texas) served as the true internal standard as performed by Binz et al. (35). The pooled QC samples yielded a 9.54% variation. The level of detection (LOD) was 0.145 pg/mg. Thirty samples contained cortisol concentrations that were unable to be detected.

### Statistical Analysis

Data were analyzed with the software R version 3.4.1 (R Core Team, Vienna, Austria) in RStudio using the car (36), plyr (37), lme4 (38), and emmeans (39) packages. Linear mixed models fit by REML were created for the numerical response of hair cortisol concentration with cattle group type (including age and color demographics), duration of growth and hair length as predictor variables using the lmer function to account for repeated measures on steers. All models were subject to a type three analyses of variance using Kenward-Roger's method. For categorical variables, estimated marginal means (or least squares means) and Tukey's adjusted pairwise comparisons were calculated. Summary statistics and data graphing were performed to check normality. Hair cortisol concentrations were base 10 logarithm transformed to achieve normality and satisfy all model assumptions. Correlation between transformed hair cortisol values and hair length values was calculated accounting for repeated measures on animals (40). All cortisol concentrations considered to be below the LOD were analyzed as values equating to 0.5^*^LOD. Three samples yielded concentrations below the LOD. Values that were out of the range of three standard deviations plus or minus the mean were considered outliers and excluded from the analysis. The data contained three hair cortisol outliers, which were removed from the analysis. Significant differences were recognized at α ≤ 0.05.

## Results

**Figure 3:**
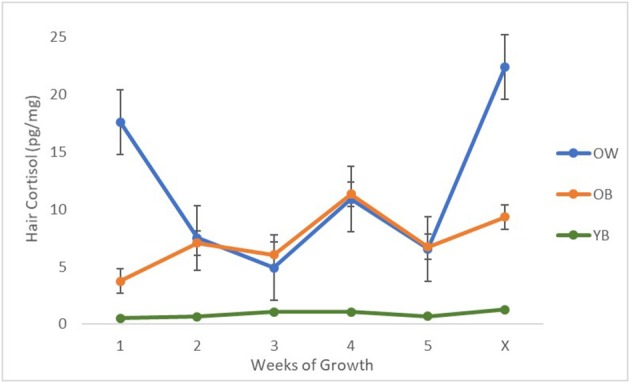
A plot between the average non-log transformed hair cortisol concentrations and the weeks of growth for the samples shaved during week 6 for each group of cattle (*N* = 18). Steers were categorized into one of three groups: OB, old with black hair (~9 years of age), *n* = 3; OW, old with white hair (~9 years of age), *n* = 3; YB, young with black hair (~2.5 years of age), *n* = 12. Weeks of growth were defined as follows: 1–5, number of weeks each sample grew between the initial shave and re-shave sample collections; X, natural, previously unshaved hair.

The age of the steers significantly impacted the hair cortisol concentration, as reported in Table 1. Older steers with both black and white hair showed higher amounts of hair cortisol than younger steers with white hair (*P* = < 0.001). No difference was observed between OB and OW (*P* = 0.99). Furthermore, the combined impact of age and color had a significant impact on hair cortisol concentrations across periods of growth (*P* < 0.001), in relation to the different areas shaved in week 6 that were allowed various durations of growth (Figure 3).

**Table 1:** Effect of cattle group in relation to age and hair color on hair cortisol concentration in beef feedlot steers (*N* = 18).

**Outcome**	***n***	**Cattle group^1^**			**Pooled** **SEM**	***P*-value**
		**OB** **(*n* = 3)**	**OW** **(*n* = 3)**	**YB** **(*n* = 12)**		
Hair cortisol (pg/mg)^2^	18	7.57^a^	10.89^a^	0.98^b^	1.15	<0.001

a, b *Within rows, values with different superscripts differ (P < 0.05)*.

1 *Steers were categorized into one of three groups: OB, old with black hair (~9 years of age), n = 3; OW, old with white hair (~9 years of age), n = 3; YB, young with black hair (~2.5 years of age), n = 12*.

2 *Means and pooled standard error are reported on the original scale, while the P-value is reported based on the base 10 logarithm transformed values that were used to obtain a normal distribution of data*.

**Figure 4:**
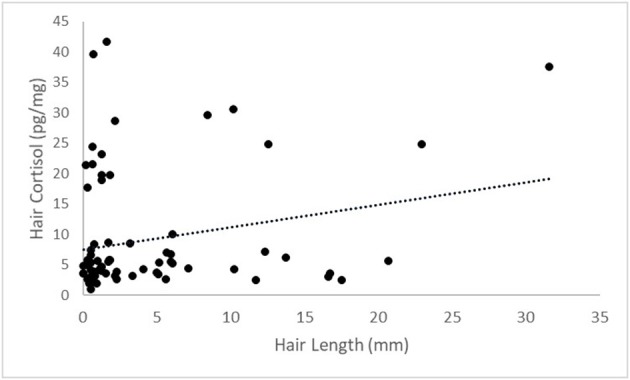
A scatterplot between base 10 logarithm transformed values for hair cortisol and hair length. A weak, positive correlation of *r* = 0.33 with a *P*-value = 0.01.

A weak, positive correlation was observed between hair cortisol concentration and hair length (*r* = 0.33, *P*-value = 0.01) (Figure 4). Though, the concentration of cortisol in the hair was not impacted by the duration of growth in terms of weeks (*P* = 0.33). Using our measured values, the average growth rate of beef cattle hair in the rump region was calculated to be 0.90 mm per week in the winter months.

## Discussion

Our reported hair cortisol concentration values ranged from 0.0725 to 31.59 pg/mg. Other studies have found similar values on both ends of the value spectrum involving beef bulls [2.31 ± 0.176 pg/mg (22); 3.52 ± 0.49 pg/mg (31)], weaned beef calves [2.36 ± 0.38 pg/mg (33)], beef bull calves [5.16 ± 1.63 pg/mg (29)], lactating dairy cows of various parities [5.7 ± 1.7 pg/mg (25)], dairy cows ranging from 3 to 17 years of age [0.69 ± 0.45 pg/mg (27)], and 2-years-old dairy cows [12.15 ± 1.85 pg/mg (24)]. Extremely high values were reported in 15-days-old heifers [114.5 ± 14.43 pg/mg (24)]. Our results encompass a large range of values, which is unique when compared to past research. Several factors may have played a role in the greater variation of our values. Cortisol concentrations have been found to be higher in the clipped hair as compared to plucked hair (22). Other factors, including animal age, hair color and sampling location have been shown to impact hair cortisol concentrations as illustrated below.

When considering the impact of cattle age on hair cortisol concentration, our results seem to be the converse of other reported values. As mentioned previously, a study with dairy cattle reported that hair samples from 2-years-old cows yielded lower cortisol concentrations than samples from 15-day-old heifer calves (24). The significant difference was mentioned to possibly be attributed to a parturition initiation pathway, known as the fetal pituitary adrenal axis, that stimulates increased serum cortisol concentrations during late stages of pregnancy (41, 42). Tallo-Parra et al. (43) found no correlations between hair cortisol concentration and age in dairy cows. Other species have shown increases in cortisol concentrations from hair samples in relation to younger ages. For example, newborn foals showed higher hair cortisol as compared to 30 or 60 days old (44). The same observations have been reported in infant and juvenile comparisons with rhesus monkeys (45) and other primates (46). These observations may be related to a reduced number of corticosteroid binding globulin concentrations present in infants, which yields greater concentrations of free plasma cortisol in humans (47). A decline in hair cortisol concentrations seems to be dependent upon age, though the time course is most likely species specific with possible increases occurring in later years of age (48). Furthermore, the drastic difference in our results may have been related to the chronic presence of a ruminal fistula for the old steers vs. the young steers. Though, all old steers in our study were in good health and not lame. Minimal research has been performed investigating the long-term impacts of ruminal or duodenal fistulas in cattle; however, two studies observed higher cortisol concentrations in cattle that were deemed to be clinically diseased compared to cows that were not (44, 49). Braun et al. (50) reported that chronically ill cattle showed elevated hair cortisol concentrations vs. acutely ill cattle. The study objectives did not involve assessing the impact of fistula presence on hair cortisol, although additional research is necessary across all species, especially cattle, to fully understand the impact of age on hair cortisol and possible stress associated with fistulas.

Hair color seems to have a reported variable impact on the overall concentration of cortisol in hair. In agreement with our findings, greater cortisol concentrations have also been observed in white cattle hair as compared to black hair (24, 25, 51). Conversely, one study reported the presence of higher cortisol concentrations in black hair in relation to white hair (26). In a study on lactating Holstein cows under heat stress conditions, no significant differences in hair cortisol levels were reported related to the hair color, yet higher cortisol levels in black coat colors as compared to white coats were observed (52). They noted that collecting hair samples that reflects the overall coat color of the animal may have an impact on measured cortisol values (52). This may especially be important when collecting hair samples from multi-colored cattle, such as roan, speckled or spotted. Nedić et al. (53) also found that hair color had no effect on hair cortisol. The effect of hair color on the concentration of hair cortisol in cattle requires more research to reach a concise conclusion as current research results are quite variable.

Our reported weak, positive correlation between hair cortisol concentration and hair length shows that there is potential for hair length to have an effect on hair cortisol concentrations. Cortisol is deposited into the hair shaft during the anagen growing phase (54). The stage of growth must also be accounted for when sampling hair. The “shave-reshave” method is often performed to ensure that enough growing hairs are collected (54). This involves shaving a specific area initially and then re-shaving the same area after an elapsed period of time to collect the regrown hair sample (54, 55). We used this method to obtain samples that consisted of growing hair of different lengths based on specific growing periods (ranging from 1 to 5 weeks). Varying hair growth profiles can exist between individuals and may need to be accounted for given certain circumstances (14). Specific areas of the body may experience different rates of hair growth. Hair cortisol concentration have been suggested to be affected by season (23), due to the changing the rate of hair growth (56). Additionally, there is great ambiguity concerning the distribution of cortisol along the length of the hair shaft. Studies involving rhesus macaques (16) and dogs (57) found no significant differences when comparing cortisol concentrations of the most proximal half and the most distal half of the hair shaft. These conclusions may not necessarily be able to be extended to the species of cattle, though if proven could impact interpretation and general hair cortisol analysis. As there are very few studies regarding the impact of length on hair cortisol, this should be a continued area of future research focus.

Our study only consisted of one general sampling area—the right rump region, though sampling location has been demonstrated to have an impact on the concentration of cortisol (22). Hair samples collected from the tail region of Angus cross beef bulls yielded higher cortisol concentrations as compared to other areas, including the hip, shoulder, neck and head (22). Cerri et al. (51) agreed with the finding that tail hair was a suitable means to measuring cortisol levels over time because of the increased amounts of cortisol observed. Furthermore, white hair showed greater cortisol concentrations in the tail as compared to the hip or top line (25). An increased rate of hair growth in the tail has been possibly attributed to the increased cortisol concentrations (22).

Finally, the average growth rate of beef cattle hair has not been extensively defined thus far. Studies with dairy cattle have been used to determine the average hair growth rate of dairy cows to be ~0.6–1 cm/month (58) with a specifically calculated rate of 0.04 ± 0.05 mm/d in the hip area (25). As previously discussed, the rate of growth has also been shown to be influenced by the location (22). Burnett et al. (25) found that hair in the hip and shoulder areas have similar growth rates, while the tail switch hair grows over ten times faster than the other areas in lactating dairy cows. Similar results were found in studies with beef cattle (58, 59). Other factors have also been proven to affect the growth rate of hair in cattle, including temperature (60, 61), photoperiod (62) and nutritional deficiencies (63). The hair growth rate may play a role in the resulting hair cortisol concentration, though additional research is necessary.

Overall, we identify the fact that these data and reported conclusions are stemming from a rather small and specific cattle population. This study involved a small sample size with fistulated animals of specific hair colors. For example, the old steer group contained animals with both black and white hair, whereas the young steer group only included animals with black hair. In addition, an animal's response to stress can be affected by previous experience and an individual's perception of the stressor (64). This further highlights the critical need for more research pertaining to cortisol quantification in the hair of beef cattle.

## Conclusion

The data indicate that hair cortisol concentrations are affected by animal age and color characteristics in beef cattle. Older steers exhibited significantly greater hair cortisol concentrations than younger steers. The duration of growth had no impact on the resulting hair cortisol concentration. Important limitations of the study, including a small, specific sample size demographic and the use of fistulated cattle, must be taken into consideration. Additional studies should be performed to assess the impact of fistula presence on the animal well-being related to chronic stress. This was a preliminary study and further research is also necessary to understand how these factors and others may impact hair cortisol concentrations in beef cattle.

## Data Availability Statement

All data sets generated for this study can be made available upon request of the authors.

## Ethics Statement

The animal study was reviewed and approved by Colorado State University Institutional Animal Care and Use Committee (IACUC).

## Author Contributions

FB, TG, TE, and LE-C had substantial contribution to the work through concept, design, analysis and/or interpretation. Additionally, all authors revised and edited the draft and approved of publication.

### Conflict of Interest

The authors declare that the research was conducted in the absence of any commercial or financial relationships that could be construed as a potential conflict of interest.
